# Prevalence of Disease and Age-Related Behavioural Changes in Cats: Past and Present

**DOI:** 10.3390/vetsci7030085

**Published:** 2020-07-06

**Authors:** Lorena Sordo, Craig Breheny, Vicky Halls, Amy Cotter, Camilla Tørnqvist-Johnsen, Sarah M. A. Caney, Danièlle A. Gunn-Moore

**Affiliations:** 1 Royal (Dick) School of Veterinary Studies and the Roslin Institute, The University of Edinburgh, Easter Bush Veterinary Campus, Roslin, Midlothian EH25 9RG, UK; craig.breheny@ed.ac.uk (C.B.); C.T.Johnsen@sms.ed.ac.uk (C.T.-J.); danielle.gunn-moore@ed.ac.uk (D.A.G.-M.); 2 International Cat Care, Place Farm, Tisbury, Wiltshire SP3 6LW, UK; vickyhallscats@aol.com; 3 Alnorthumbria Veterinary Group, Wagonway Road, Alnwick, Northumberland NE661QQ, UK; Amy.Cotter@alnorthumbriavets.co.uk; 4 Vet Professionals Ltd., Midlothian Innovation Centre, Pentlandfield, Roslin EH25 9RE, UK; sarah@vetprofessionals.com

**Keywords:** normal ageing, elderly cats, behavioural changes, prevalence of disease

## Abstract

(1) Background: age-related changes in behaviour and health may be thought of as “normal” ageing; however, they can reflect under-diagnosed, potentially treatable, conditions. This paper describes the prevalence of age-related behavioural changes and disease in two UK cat populations at separate time-points. (2) Methods: owners of cats aged ≥11 years completed questionnaires in 1995 (cohort 1: *n* = 1236), and from 2010–2015 (cohort 2: *n* = 883). (3) Results: the most important behavioural changes in these cats were increased affection towards their owners (reported by 51.9% in 1995; 35.8% in 2010–2015), increased vocalisation (63.5%; 58.9%, respectively), particularly at night (32%; 43.6%), and house-soiling (29.3%; 55.8%). Most (79.4%; 81%) of the cats had visited a veterinary surgeon since becoming 11 years old. The main reasons, aside from vaccinations, were dental disease, renal disease and lower urinary tract disorders in 1995, and dental disease, renal disease and hyperthyroidism in 2010–2015. All major diagnoses were reported significantly more frequently in 2010–2015 than in 1995; behavioural changes were variably associated with these diseases. (4) Conclusion: elderly cats display age-related behavioural changes and develop diseases that may be under-diagnosed. Veterinarians need to ask owners about these behavioural changes, as they may signify manageable conditions rather than reflect “normal” ageing.

## 1. Introduction

With improvements in nutrition and veterinary medicine, the life expectancy of pet cats is increasing and the number of elderly cats seen by veterinary surgeons is growing [1,2,3,4]. Approximately 30–50% of pet cats in the United States are now believed to be of seven years of age or older [5]; 13% of the cats are aged 12 years or older [5]; and there was a 15% increase in the number of cats over 10 years of age seen at veterinary clinics from 1983 to 1993 [6]. A study of insured cats in Sweden confirmed this trend, with 58% of Birman cats surviving to 12.5 years of age during 1999 to 2002, whereas between 2003 and 2006 this increased to 68% survival to this age [7]. Similarly, with Siamese cats, survival during the two periods increased from 33% to 42% [7]. In the UK, there are currently believed to be approximately 2.5 million ‘senior’ cats [4,5,6,7,8]. The management of these individuals is becoming increasingly important for small animal veterinary practitioners, as they constitute a large population of patients. 

Details about age-related behavioural changes and common diseases seen in elderly cats have been reported previously [9,10]. Recognising behavioural changes that are common in old age is important as they can result from systemic diseases, intracranial pathology or neuronal degeneration [9,10]. Behavioural changes may develop before any other signs of illness, highlighting the importance of their early recognition. Some of the most common age-related behavioural changes include house-soiling, excessive vocalisation, altered sleep cycle, and disorientation [9,10]. The most common medical problems seen in elderly cats include mobility disorders (e.g., osteoarthritis); endocrine disease (e.g., diabetes mellitus, hyperthyroidism); hypertension; neurological disease (e.g., neoplasia and sensory deficits); organ failure (e.g., kidney, heart, and liver failure); and cognitive dysfunction syndrome [9,10]. Alongside this, geriatric patients are more likely to be presented with co-morbidities [9,10], resulting in the need for polypharmacy, which can make their management challenging, although rewarding, nonetheless. 

Veterinarians can suggest many ways that owners can help their elderly cats. These include showing the owners what behaviours are normal and which ones may be a cause for concern and need to be investigated, as well as suggesting ways of adapting their homes and lifestyles to best accommodate their ageing cats to support a long and comfortable life [4]. In order to give the best advice to owners, veterinarians need to know how cats change with age. While they can learn a great deal by reading reference texts and looking at clinic records these can only help so far, they also need to listen to the cats’ owners. This is because owners can identify subtle changes in their pet (without necessarily understanding the significance of them). Unfortunately, the changes may not be apparent in a veterinary clinic because the stress of being there leads the cat to alter its behaviour. Being able to recognise abnormal clinical signs relies on understanding what changes are within the realms of normal ageing, and what are not. This paper provides evidence about the most common behavioural changes and diseases seen in elderly cats, according to their owners, in two large groups of pet cats in the UK. These findings will enable both cat owners and veterinarians to recognise these changes, and to differentiate between normal and abnormal; by doing this, specific cats’ requirements can be met, improving their quality of life and welfare.

The first aim of this paper was to describe the most common behavioural changes as well as the most prevalent diseases seen in elderly cats: this was performed by assessing two different cohorts of cats, one in 1995 and another one from 2010–2015. The investigation then assessed changes in the prevalence of these behaviours over the intervening 15 to 20 years.

## 2. Materials and Methods 

Data were collected through surveys of two different cohorts of owners of elderly cats. The first questionnaire was available in 1995, for six months, while the second was available online, from 2010–2015.

The questionnaire in 1995 (“The Elderly Cat Survey”) was designed by one of the authors (VH) and consisted of two-pages containing 24 open and closed questions (Supplementary material 1). The open questions were included to gain an insight into the quality of the relationship between the owner and the cat. The survey was promoted in regional (The West Briton) and national newspapers (The Daily Telegraph), and a cat magazine (“All About Cats”); owners of a cat of 11 years of age or older were asked to forward a stamped self-addressed envelope (SAE) if they would like to be sent a copy of the questionnaire to complete and return. They were told that their data would be included in a study that would be submitted as part of a degree and potentially published.

In 2010, a second questionnaire was designed to gain more up-to-date data to see how things had changed in the intervening years. However, the SAE method of data collection was considered outdated and insecure, so the second questionnaire was available online. Not all questions included in the first questionnaire were included in the second, as they had been difficult to analyse; new questions were added to try to improve clarity. This questionnaire was available on the Vet Professionals website (http://www.vetprofessionals.com/) from 2010 to 2015; this company runs numerous online questionnaire studies, collecting, collating, and storing the data. This questionnaire consisted of 44 questions, of a similar design to that used in 1995, combining open and closed questions (Supplementary material 2). The questionnaire was accessible worldwide; but only UK data was analysed further. Owners were asked to reply for their own cat of 11 years of age or older.

Owners did not necessarily answer every question for every cat, so the analysis was completed on the data that were available for each individual question. Frequencies are reported with numbers and percentages of responses received. As the online questionnaire was available for a number of years, some cats had multiple entries, some fully and others only partially completed. If more than one copy was submitted, only the first completed questionnaire was included. 

The questionnaires from 1995 and 2010–2015 were analysed separately using Minitab 17 Statistical Software (2010) (Minitab Inc. Coventry, UK). Data from both questionnaires was treated as categorical to determine whether a certain behaviour was associated with age by performing ordinal, nominal or binary logistic regression, depending on the number and/or order of the categories for each variable. For all regression models, the different behaviours and/or diseases were adjusted for age. Default terms in the model with no interactions were used, with two-sided 95% confidence intervals and a significance level of α = 0.05. Goodness-of-fit tests were calculated by the Pearson’s chi-square method for both ordinal and nominal logistic regression and by the Hosmer–Lemeshow method for the binary logistic regression. This compared cats within each group and time-period; the cats aged 11–15 years old, 16–19 years and those equal or greater than 20 years old in 1995 were compared with each other, while cats aged 11–13 years old, 14–16 years, 17–19 years, and those equal or greater than 20 years old in 2015 were compared with each other. Two-sample z-test were performed to determine whether there were any differences between the populations of both surveys. 

Binary logistic regressions were performed, as described above, on the data from both questionnaires, separately, to assess whether there was a difference between different diseases the owners reported and the age of their cats. 

Finally, Pearson’s chi-square and Fisher’s exact tests were performed to determine associations between the different behaviours and disease, where each of the behaviours were compared against each disease, separately. For the 2010–2015 questionnaire, similar responses were grouped together; for this, “a lot less” and “little less” responses were grouped into the “Decrease” group; “little more” and “much more” responses were grouped into the “Increase” group; and “Same” answers remained as they were.

Ethical approval by the Human Ethical Review Committee (HERC) was not requested for this study as it had not been introduced at the time the study started (1994), or when it was repeated (2009). However, the participants of both surveys expressly stated that by submitting their replies they were giving permission for the data to be used for research and publication. In addition, all data were stored and analysed anonymously, complying with current General Data Protection Regulations (GDPR) with regards to storing personal data and participation consent.

## 3. Results

### 3.1. Demographics

A total of 1236 responses were analysed from the data collected in 1995; cats were aged 11 to 15 years old (61.3%), 16 to 19 years old (32.6%), and of 20 years old or more (6.1%). Most cats (80.3%) were non-pedigree (i.e., domestic short or long hair) with 19.7% being purebred cats. Just over half (55.1%) were neutered females, 44.4% of cats were neutered males, few were entire males (0.5%) and none were entire females. Most (68.1%) cats had lived in their household since they were kittens.

From 2010–2015, a total of 883 responses were analysed; cats were aged 11 to 13 years old (34.7%), 14 to 16 years (39.3%), 17 to 19 years (21.7%) and 20 years old or more (4.3%). Most cats (84.7%) were non-pedigree, with 15.3% being purebred. Half of the cats were neutered females (50.7%), 44.5% were neutered males, 3.1% were entire males and 1.7% were entire females. 

The only significant difference in the demographics between the two surveys was the proportion of cats over 20 years old; a greater proportion were over 20 years of age in the 1995 cohort (*p* = 0.05).

### 3.2. Behavioural Changes

Both surveys showed that cats displayed different age-related behavioural changes, with some of them being reported by the owners as increased (Table 1) or decreased (Table 2). The complete list with statistical test results of the logistic regressions can be found in Supplementary material 3.

#### 3.2.1. Vocalisation During the Day

**Figure 1:**
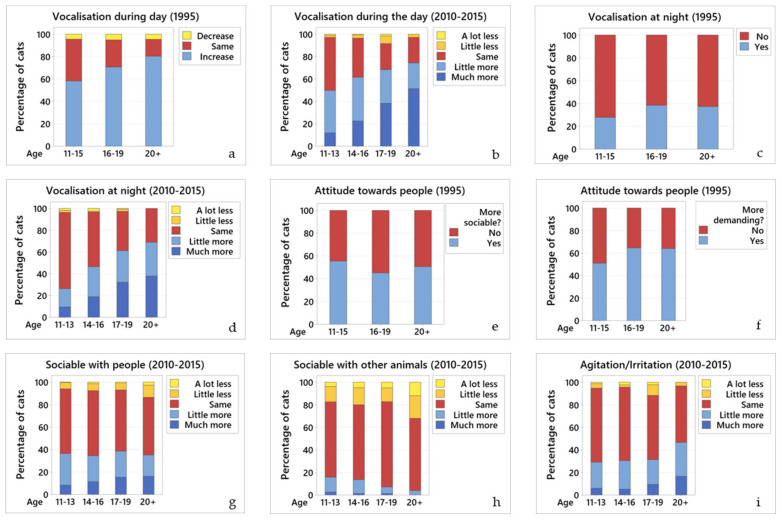
Changes in the different behaviours across age groups. Comparative graphs showing changes in behaviour across age groups for (**a**) vocalisation during the day (1995); (**b**) vocalisation during the day (2010–2015); (**c**) vocalisation at night (1995); (**d**) vocalisation at night (2010–2015); (**e**) attitude towards people—more sociable (1995); (**f**) attitude towards people—more demanding (1995); (**g**) sociability with people (2010–2015); (**h**) sociability with other animals (2010–2015); and (**i**) agitation/irritation (2010–2015). In all graphs of this panel, blue colours represent an increase in the particular behaviour, yellow represent a decrease, and red represent no change.

Data from 1995: many (63.5%; 734/1156) cats vocalised more than before, 31.8% (368/1156) vocalised the same amount, and 4.7% (54/1156) less. Owners reported that while 6.3% (77/1236) of their cats vocalised for food, the majority (93.7%; 1159/1236) vocalised during the day for no apparent reason. There was a significant relationship between ageing and vocalisation during the day; older cats tended to vocalise more often, without apparent reason (OR = 0.60, 95% CI 0.49 to 0.75, *p* < 0.001) (Figure 1a). Owners reported that their cats started vocalising more at a mean of 13 years of age. 

Data from 2010–2015: most cats (58.9%; 488/828) vocalised more than they used to; 36.7% (304/828) vocalised the same amount as before, and 4.4% (36/828) vocalised less. There was a significant relationship between ageing and increased vocalisation during the day; older cats tended to vocalise more (OR = 0.62, 95% CI 0.53 to 0.72, *p* < 0.001) (Figure 1b).

#### 3.2.2. Vocalisation at Night

Data from 1995: a third (32%; 393/1233) of cats vocalised at night for no apparent reason (“night calling”). Most of these cats (84.5%; 246/291) started this behaviour when they were over 10 years old. There was a significant relationship between ageing and increased vocalisation at night; older cats tended to vocalise more (OR = 1.40, 95% CI 1.15 to 1.70, *p* = 0.001) (Figure 1c).

Data from 2010–2015: over half (53.2%; 349/656) of the cats vocalised at night with the same frequency as they had before, 43.6% (286/656) vocalised more than before and only 3.2% (21/656) vocalised less. There was a significant relationship between ageing and increased vocalisation at night; older cats tended to vocalise more (OR = 0.52, 95% CI 0.43 to 0.62, *p* < 0.001) (Figure 1d).

#### 3.2.3. Attitude Towards People

Data from 1995: owners were asked a yes or no question as to whether their cat was more social with them than it had been previously; 51.9% (641/1236) of owners replied yes (OR = 0.77, 95% CI 0.64 to 0.93, *p* = 0.007). However, when looking at the three age groups, this increase in sociability was slightly less marked in the cats aged 16–19 years, although they were still more social than earlier in life (Figure 1e). 

When asked if their cats were more demanding than previously, 56.1% (694/1236) of owners said yes; older cats tended to be more demanding (OR = 1.51, 95% CI 1.25 to 1.83, *p* < 0.001) (Figure 1f).

Data from 2010–2015: owners were asked whether their cat was more social with them than previously, then selected from options: increase, no change, or decrease. Over half (56.9%; 500/879) of the cats were as sociable with people as they had been before, 35.8% (315/879) were more sociable than before, and 7.3% (64/879) were less sociable. While 35.8% of cats were more sociable than before, there was no significant relationship between ageing and how sociable cats are, as the cats continued to be as sociable as before from 11 years onwards (Figure 1g). The 2015 questionnaire did not ask whether or not cats had become more demanding.

#### 3.2.4. Sociable with Other Animals

Data from 1995: half of the cats (56%; 608/1087) shared their home with other animals (i.e., dogs, cats, other small animals). Most (62.5%; 377/604) were as sociable with other animals in the household as before; 12.3% (74/604) were less sociable and 25.2% (153/604) were more sociable. There was no statistical relationship between ageing and how sociable cats are to other animals in the household. 

Most (82.4%; 1018/1236) of the cats used to be territorial. Of these, 39.8% (405/1018) were still showing territorial aggression. In contrast, 34.4% (350/1018) were more tolerant of other cats in their territory. There was no significant association between ageing and territorial aggression.

Data from 2010–2015: most (67.9%; 476/701) cats were as sociable with other animals within their household as before; 19.1% (134/701) were less sociable and 13% (91/701) were more sociable. There was a significant relationship between ageing and how sociable the cats were to other animals within their household; cats tended to become less sociable as they get older (OR = 1.24, 95% CI 1.03 to 1.50, *p* = 0.022) (Figure 1h).

#### 3.2.5. Agitation/Irritation for No Apparent Reason

Data from 1995: no data from the 1995 questionnaire.

Data from 2015: most (74%; 653/881) cats displayed agitation/irritation for no apparent reason some of the time. There was no relationship between ageing and agitation/irritation (Figure 1i).

#### 3.2.6. Changes in Appetite

Data from 1995: half (51.2%; 632/1235) of the cats were fed on demand, with fewer being fed twice daily (34.5%; 426/1235), three times daily (13%; 161/1235) or once daily (1.3%; 16/1235); 56.9% (701/1233) ate the same amount as before, 23.5% (290/1233) ate less, and 19.6% (242/1233) ate more. The relationship between ageing and appetite was statistically significant; older cats tended to eat less (OR = 1.48, 95% CI 1.24 to 1.77, *p* < 0.001).

**Figure 2:**
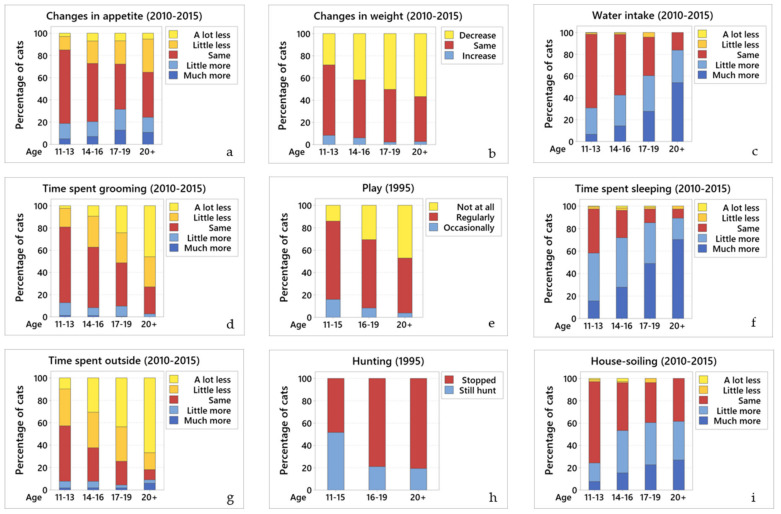
Changes in the different behaviours across age groups. Comparative graphs showing changes in behaviour across age groups for (**a**) changes in appetite (2010–2015); (**b**) changes in weight (2010–2015); (**c**) water intake (2010–2015); (**d**) grooming (2010–2015); (**e**) play behaviour (1995); (**f**) amount of time sleeping (2010–2015); (**g**) outside access (2010–2015); (**h**) hunting behaviour (1995); and (**i**) house-soiling (2010–2015). In all graphs of this panel, blue colours represent an increase in the particular behaviour, yellow represent a decrease, and red represent no change.

Data from 2010–2015: over half (54.2%; 475/876) of the cats ate the same as before, 23.5% (206/876) ate less, and 22.3% (195/876) ate more. There was no significant association between ageing and changes in the appetite between the age groups (Figure 2a).

#### 3.2.7. Changes in Weight

Data from 1995: no data from the 1995 questionnaire.

Data from 2010–2015: the majority (70.7%; 624/882) of owners knew their cat’s weight. Most cats (88.1%; 539/612) weighed 2–4 kg, while 11.9% (73/612) weighed 4–6 kg. Over half (54.5%; 464/852) had not changed weight, 39.4% (336/852) had lost weight, and 6.1% (52/852) gained weight. There is a significant relationship between age and changes in body weight; older cats tended to lose weight (OR = 1.57, 95% CI 1.34 to 1.84, *p* < 0.001) (Figure 2b).

#### 3.2.8. Water Intake

Data from 1995: half of the cats (58.2%; 620/1066) drank more than before. There was a significant association between age and water intake; older cats tended to drink more (OR = 2.57, 95% CI 2.04 to 3.23, *p* < 0.001).

Data from 2010–2015: half of the cats (53.5%; 469/876) drank the same amount as before, 2.2% (19/876) drank less, and 44.3% (388/876) drank more. There was a significant relationship between age and water intake; older cats tended to drink more water (OR = 0.50, 95% CI 0.43 to 0.59, *p* < 0.001) (Figure 2c).

#### 3.2.9. Grooming

Data from 1995: the majority (75.2%; 929/1234) of cats still groomed frequently, 22.9% (282/1234) only groomed occasionally and 1.9% (23/1234) had stopped grooming. Age was statistically associated with grooming; older cats tended to groom less frequently (OR = 2.47, 95% CI 1.35 to 4.53, *p* = 0.003). 

Data from 2010–2015: over half (54.5%; 478/873) of the cats groomed the same amount as before, 35.6% (309/873) groomed less, and 9.9% (86/873) groomed more. There was a significant relationship between age and the amount of time a cat spends grooming; older cats tended to groom less (OR = 2.11, 95% CI 1.81 to 2.47, *p* < 0.001) (Figure 2d).

#### 3.2.10. Play

Data from 1995: most (72.6%; 897/1236) cats used to play. Of the cats that still played, 65.7% (599/912) still played occasionally, 12.9% (118/912) played regularly, and 21.4% (195/912) had stopped playing. There was a statistical relationship between age and play; older cats tended to play less (OR = 3.91, 95% CI 2.50 to 6.12, *p* = 0.012) (Figure 2e).

Data from 2010–2015: no data from this questionnaire.

#### 3.2.11. Amount of Time Sleeping

Data from 1995: over half (54.8%; 676/1233) of the cats slept 50–75% of the day, 39.5% (487/1233) slept more than 75% of the day, and 5.7% (70/1233) slept less than 50% of the day. There was a significant relationship between ageing and the time spent asleep; older cats tended to sleep more (OR = 0.41, 95% CI 0.26 to 0.67, *p* < 0.001).

Data from 2010–2015: the majority of cats (70.6%; 619/877) slept more than before, 26.3% (231/877) slept the same amount, and 3.1% (27/877) slept less. There was a significant relationship between ageing and the amount of time a cat sleeps; older cats tended to sleep more (OR = 0.47, 95% CI 0.41 to 0.55, *p* < 0.001) (Figure 2f).

#### 3.2.12. Willingness to Go Outside

Data from 1995: most cats (97%; 1188/1225) had outdoor access, although none of those aged 20 years old or older went outside. Over half (52.2%; 639/1225) of the cats went outside less than before, 41% (502/1225) went outside the same amount and 6.8% (84/1225) went outside more. There was a significant relationship between ageing and the drive to go outside; older cats tended to go out less (OR = 1.92, 95% CI 1.58 to 2.34, *p* < 0.001).

Data from 2010–2015: most (83.5%; 737/882) cats had access outside; however, more than half (58.5%; 441/754) went out less than before, 34.2% (258/754) went out the same amount, and 7.3% (55/754) went out more. Of the cats that did go outside: 36% (262/727) were outside for less than 1 h, 47.3% (344/727) for 1–6 h, 11.2% (81/727) for 6–12 h, 4.8% (35/845) for 12–24 h, and 0.7% (5/727) were outside all day (i.e., 24 h a day). There was a significant relationship between ageing and the want to go outside; older cats tended to spend less time outside (OR = 2.08, 95% CI 1.76 to 2.45, *p* < 0.001) (Figure 2g).

#### 3.2.13. Hunting

Data from 1995: most (78.5%; 950/1210) cats used to hunt; of these, 39.4% (374/950) still hunt. There was a statistical association between ageing and hunting behaviour; older cats tended to hunt less (OR = 0.32, 95% CI 0.24 to 0.41, *p* < 0.001) (Figure 2h).

Data from 2010–2015: no data from this questionnaire.

#### 3.2.14. House-Soiling

Data from 1995: despite 55.5% (685/1235) of cats having access to a litterbox, 29.3% (362/1234) experienced house-soiling. There was a statistical association between ageing and house-soiling; older cats tended to house-soil more (OR = 2.29, 95% CI 1.87 to 2.79, *p* < 0.001).

Data from 2010–2015: despite most (82.9%) (732/883) cats having access to a litterbox, 55.8% (492/882) experienced house-soiling. There was a significant relationship between ageing and house-soiling; older cats tended to house-soil more (OR = 0.55, 95% CI 0.45 to 0.67, *p* < 0.001) (Figure 2i).

### 3.3. Visit to Veterinarians and Diseases Diagnosed

Most (80.5%) (981/1218) cats visited a veterinary clinic for various reasons in 1995. The most common reasons were routine booster vaccinations (32.4%; 263/813), dental disease (15.4%; 125/813), renal disease (6.6%; 54/813), and lower urinary tract disorders (5.4%; 44/813). 

Of the cats that visited veterinary clinics in 2010–2015, 80.2% (708/882) had their weight checked, 75.9% (670/883) went for reasons other than vaccination, 66.3% (585/882) for vaccination, 65.5% (577/882) had blood tests, 39.2% (346/883) had urine analysed, and 24.5% (216/883) had their blood pressure measured (options were not exclusive as many cats had a number of reasons for an individual consultation e.g., vaccination and blood pressure assessment, or vaccination during which significant problems were identified). The most common diseases for which owners took their cat to the clinic were dental disease (14.9%; 100/669), renal disease (11.3%; 76/669), and hyperthyroidism (10.8%; 72/669).

### 3.4. Prevalence of Disease

Owners were asked what diseases their cats lived with; a summary of the prevalence of these different diseases can be found in Table 3.

#### 3.4.1. Kidney Disease

Only 6.1% (75/1236) of cats were reported to have kidney disease in 1995. In contrast, almost a quarter (23.1%; 204/882) of cats were reported to have kidney disease in 2010–2015. Older cats were more likely to develop kidney disease in both surveys (OR = 1.79, 95% CI 1.27 to 2.53, *p* = 0.001; and OR = 2.04, 95% CI 1.67 to 2.48, *p* < 0.001, respectively).

#### 3.4.2. Blindness

Few (2.5%; 31/1236) cats were reported to be blind in 1995. Similarly, blindness was reported in only 10.5% of cats (93/882) in 2010–2015, with older cats being more likely to develop blindness (OR = 2.21, 95% CI 1.71 to 2.85, *p* < 0.001).

#### 3.4.3. Lower Urinary Tract Infections

Only a few cats from both surveys were reported to have lower urinary tract infections: 0.8% (10/1236) in 1995, and 10.4% (92/881) in 2010–2015.

#### 3.4.4. Hyperthyroidism

Few (1.2%; 15/1236) cats were reported to have hyperthyroidism in 1995. Data from 2010–2015 showed that hyperthyroidism was reported in only 13.9% of cats (123/883), with older cats being more prone to develop hyperthyroidism (OR = 1.63, 95% CI 1.30 to 2.05, *p* < 0.001).

#### 3.4.5. Diabetes Mellitus

Only 0.7% of cats (9/1236) and 2.3% (9/1236) were reported to have diabetes mellitus, in 1995 and 2010–2015, respectively. 

#### 3.4.6. Arthritis

Data from 1995 showed that only a few (6.6%; 81/1236) cats were reported to have arthritis. In contrast, over a third (35.9%; 317/883) of cats were reported to have arthritis in 2010–2015. Older cats were more likely to develop arthritis in both surveys (OR = 1.51, 95% CI 1.07 to 2.12, *p* = 0.02; and OR = 1.93, 95% CI 1.61 to 2.32, *p* < 0.001, respectively).

#### 3.4.7. Heart Disease

Few (0.8%; 10/1236) cats were reported to have heart disease in 1995. Similarly, only 4.3% (38/882) cats were reported to have heart disease in 2010–2015. Older cats were more likely to develop heart disease (OR = 1.49, 95% CI 1.03 to 2.15, *p* = 0.036).

#### 3.4.8. Deafness

Deafness was reported in 4.6% (57/1236) cats in 1995, and 12.5% (110/882) of cats in 2010–2015. Results from both surveys showed that older cats were more likely to develop deafness (OR = 4.42, 95% CI 3.00 to 6.52, *p* < 0.001; and OR = 3.19, 95% CI 2.46 to 4.14, *p* < 0.001).

#### 3.4.9. Dental Disease

Only 0.6% (8/1226) cats were reported to have dental disease in 1995. In contrast, dental disease was reported in over a third (31.3%; 276/883) of cats in 2010–2015. Older cats were more likely to develop dental disease (OR = 1.25, 95% CI 1.05 to 1.48, *p* = 0.009).

**Figure 3:**
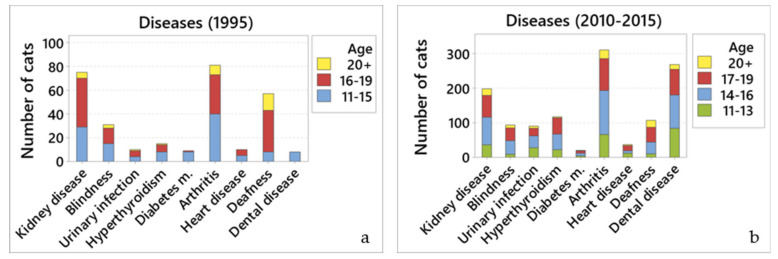
Main diseases reported by cat owners. Comparison of the number of cats reported to have different diseases by age groups for (**a**) 1995; and (**b**) 2010–2015.

Summary data relating to visits to veterinarians and diseases diagnosed for both 1995 and 2010–2015 are shown in Figure 3.

### 3.5. Associations Between Behaviours and Disease 

Pearson’s Chi Square and Fisher’s Exact Tests demonstrated several associations between the different behaviours and diseases. Only Statistically Significant Results are Shown.

#### 3.5.1. Kidney Disease

In 1995, cats reported as having kidney disease showed statistically significant associations with decreased appetite (X^2^ = 23.838, *p* < 0.001); drinking more water (X^2^ = 29.96, *p* < 0.001); reduced time spent grooming (X^2^ = 21.15, *p* < 0.001); increased vocalisation during the day (*p* = 0.01); and increased house-soiling (X^2^ = 5.54, *p* = 0.019). 

In 2010–2015, cats showed associations with kidney disease and decreased appetite (X^2^ = 97.92, *p* < 0.001; drinking more water (X^2^ = 168.55, *p* < 0.001); increased time spent sleeping (X^2^ = 24.91, *p* < 0.001; increased vocalisation during the day (X^2^ = 21.48, *p* < 0.001), increased vocalisation at night (X^2^ = 20.76, *p* < 0.001; reduced time spent grooming (X^2^ = 38.65, *p* < 0.001); weight loss (X^2^ = 67.12, *p* < 0.001); increased house-soiling (X^2^ = 11.37, *p* = 0.003); increased agitation/irritation (X^2^ = 11.18, *p* = 0.004); less willingness to go outside (X^2^ = 9.26; *p* = 0.010); decreased sociability with other animals (X^2^ = 6.76, *p* = 0.034); and more sociable with people (X^2^ = 6.76, *p* = 0.034).

#### 3.5.2. Blindness

In 1995, cats reported as being blind showed statistically significant associations with increased sociability with people (X^2^ = 6.68, *p* = 0.04).

In 2010–2015, cats showed associations with blindness and increased water intake (X^2^ = 15.728, *p* < 0.001); reduced time spent grooming (X^2^ = 38.40, *p* < 0.001); increased house-soiling (X^2^ = 14.99, *p* = 0.001); increased vocalisation at night (X^2^ = 14.90, *p* = 0.001); increased agitation/irritation (X^2^ = 10.68, *p* = 0.005); weight loss (X^2^ = 10.23, *p* = 0.006); increased time spent sleeping (X^2^ = 9.93, *p* = 0.007); increased sociability with people (X^2^ = 8.15, *p* = 0.017); decreased sociability with other animals (X^2^ = 6.76, *p* = 0.034); and reduced appetite (X^2^ = 6.24, *p* = 0.04).

#### 3.5.3. Lower Urinary Tract Infections

In 1995, cats reported as having lower urinary tract infections showed no statistically significant associations with any of the behaviours reported. 

In contrast, in 2010–2015 cats showed associations with lower urinary tract infections and reduced appetite (X^2^ = 13.13, *p* =0.001); increased water intake (X^2^ = 7.39, *p* = 0.02); and increased house-soiling (X^2^ = 3.73, *p* = 0.05).

#### 3.5.4. Hyperthyroidism

In 1995, cats reported as having hyperthyroidism showed statistically significant associations with being more demanding of attention (X^2^ = 5.74, *p* = 0.02); and increased water intake (X^2^ = 3.78, *p* = 0.05). 

In 2010–2015, cats showed associations with hyperthyroidism and increased vocalisation during the day (X^2^ = 16.02, *p* < 0.001); increased water intake (X^2^ = 17.92, *p* < 0.001); reduced appetite (X^2^ = 39.59, *p* < 0.001); weight loss (X^2^ = 39.41, *p* < 0.001); increased vocalisation at night (X^2^ = 7.09, *p* = 0.019); reduced time spent grooming (X^2^ = 7.31, *p* = 0.026); and less willingness to go outside (X^2^ = 6.78, *p* = 0.034).

#### 3.5.5. Diabetes Mellitus

Cats reported as having diabetes mellitus showed no statistically significant associations with any of the behaviours reported in the questionnaires.

#### 3.5.6. Arthritis

In 1995, cats reported as having arthritis showed statistically significant associations with increased time spent sleeping (X^2^ = 17.93, *p* < 0.001); reduced willingness to go outside (X^2^ = 14.30, *p* = 0.001); increased house-soiling (X^2^ = 6.68, *p* = 0.01); and increased vocalisation during the day (X^2^ = 5.77, *p* = 0.016).

In 2010–2015, cats showed associations with arthritis and increased time spent sleeping (X^2^ = 27.11, *p* < 0.001); less willingness to go outside (X^2^ = 28.51, *p* < 0.001); reduced appetite (X^2^ = 21.04, *p* < 0.001); reduced time spent grooming (X^2^ = 56.38, *p* < 0.001); weight loss (X^2^ = 15.05, *p* = 0.001); increased water intake (X^2^ = 25.77, *p* < 0.001); increased sociability with people (X^2^ = 12.68, *p* = 0.002); decreased sociability with other animals (X^2^ = 12.15, *p* = 0.002); increased agitation/irritation (X^2^ = 10.29, *p* = 0.006); increased vocalisation at night (X^2^ = 8.09, *p* = 0.018); and increased vocalisation during day (X^2^ = 6.86, *p* = 0.032).

#### 3.5.7. Heart Disease

In 1995, cats reported as having heart disease showed no statistically significant associations with any of the behaviours reported.

In contrast, in 2010–2015 cats showed associations with heart disease and reduced time spent grooming (X^2^ = 7.71, *p* = 0.021); and weight loss (X^2^ = 6.55, *p* = 0.038).

#### 3.5.8. Deafness

In 1995, cats reported as being deaf showed statistically significant associations with increased time spent sleeping (X^2^ = 17.59, *p* < 0.001); reduced time spent grooming (X^2^ = 9.75, *p* = 0.008); increased water intake (X^2^ = 6.86, *p* = 0.009); reduced appetite (X^2^ = 8.75, *p* = 0.013); reduced willingness to go outside (X^2^ = 7.96, *p* = 0.02); increased vocalisation at night (X^2^ = 5.17, *p* = 0.023); increased house-soiling (X^2^ = 4.70, *p* = 0.030); and increased vocalisation during the day (*p* = 0.044).

In 2010–2015, cats showed associations with deafness and increased time spent sleeping (X^2^ = 21.16, *p* < 0.001); increased vocalisation at night (X^2^ = 46.36, *p* < 0.001); increased vocalisation during day (X^2^ = 20.81, *p* < 0.001); increased water intake (X^2^ = 25.77, *p* < 0.001); reduced time spent grooming (X^2^ = 26.58, *p* < 0.001); reduced appetite (X^2^ = 14.56, *p* = 0.001); weight loss (X^2^ = 14.30, *p* = 0.001); less willingness to go outside (X^2^ = 12.96, *p* = 0.002); and decreased sociability with other animals (X^2^ = 7.25, *p* = 0.027).

#### 3.5.9. Dental Disease

In 1995, cats reported as having dental disease showed no statistically significant associations with any of the behaviours reported. 

In contrast, in 2010–2015 cats showed associations with dental disease and reduced time spent grooming (X^2^ = 10.67, *p* = 0.005); decreased sociability with other animals (X^2^ = 8.63, *p* = 0.013); and reduced appetite (X^2^ = 7.48, *p* = 0.024).

### 3.6. Owner’s Feelings about Their Cats

When asked if they still gained as much pleasure from having their cat as they did when it was younger, almost all (1058/1103, 95.9%) of the owners in 1995 said yes. This question was not asked in 2010–2015.

## 4. Discussion

These two questionnaire surveys generated extensive information about the changes in behaviour and health of two large populations of elderly pet cats in the UK, as reported by their owners. They showed that many aspects of the cats’ lives had changed as they aged, from general behavioural changes (e.g., vocalisation, eating, drinking, urinating/defecating, sleeping, grooming, playing, and hunting), through to changes in social interactions with their owners and other pets (cats and dogs) within and out-with the household. They also showed that many of the cats were living with chronic ill health. Surprisingly, there were few differences between the two time periods as related to behavioural changes. This was unexpected since it might be presumed that cat care would have changed over the intervening 15 to 20 years, so improving cat behaviour. That said, all major diagnoses were detected significantly more frequently in 2010–2015 than 1995, most notably kidney disease, hyperthyroidism, arthritis, and dental disease, which may have played a role in changing affected cats’ behaviour. This may have been the case, as shown by the disease and behaviour associations demonstrated. Despite their cat’s deleterious behaviours (e.g., excessive vocalisation and house-soiling), and their chronic ill-health, the owners were as fond of their elderly cats as previously, and still gained a great deal of pleasure from living with them. 

Many general behaviours changed as the cats aged. Common changes included eating less, drinking more, losing weight, and house-soiling, despite having access to an indoor litterbox. In addition, many of the cats had either reduced or stopped grooming, went outside less, played and hunted less, and spent more time sleeping. 

There are a number of reasons why older cats may eat less, which almost a quarter of them were doing in this study (23.5% in both surveys). This could be due to physiological decline and/or physical disease (reduced senses of smell or taste, dental disease, inflammatory bowel disease, neoplasia, etc.). Alternatively, it could result from a reduced calorie requirement associated with decreased physical activity, which is common in elderly cats; or follow behavioural reinforcement, inadvertently induced by owners who try to tempt their cat by providing ever more palatable meals instead of their regular biscuits and wet food. In this case, cats may ignore their biscuits and normal wet food, as they have learnt that, by doing so, more palatable and higher calorie food will be provided. As in other studies [12], medical conditions that can affect appetite were commonly reported in the cats in the current paper, particularly dental and renal disease, and arthritis. Since weight loss was reported, it is unlikely that the loss of appetite was simply behavioural, hence physiological decline and/or physical disease likely played a major role. 

Approximately 20% of cats were reported to have an increased appetite (19.6% then 22.3%). An increase appetite in older cats could be genuine, associated with pathological causes of polyphagia, such as hyperthyroidism and diabetes mellitus, or result from the increased food consumption needed as digestive and absorptive abilities decline with age [3,13]. However, it is also possible that some of the cats did not have an increased appetite, they were simply spending more time in the house, so their eating was more evident, or they were observed spending more time at their food bowl because mouth pain necessitated slower eating. 

Since geriatric cats have a greater risk of being underweight (30–50%) than overweight (<20%) [13,14,15,16,17,18] and significant weight loss is often the first sign of serious disease [3,15,19], it is important that veterinary surgeons monitor cat body weight, percentage weight change, and body condition score closely, and ask owners to watch their cat’s weight to ensure that any reduction does not go un-investigated. Calculating the percentage weight change since the previous visit is an excellent way of monitoring weight change in cats [3]. While data was not collected in 1995, 70.7% of owners from 2010–2015 knew their cat’s weight, with 39.4% reporting weight loss. 

The effect of diet on the health of cats has been widely studied. Research has shown that feeding cats with dry food may aid dental health and reduce periodontal disease [20,21]; however dry food has also been associated with obesity [15,22]. It is possible that diet may have played a role in the development of disease and/or behavioural changes in the present study; however, that information was not collected in either of the questionnaires, which is a limitation of the study. It is plausible that there could have been differences in the cats’ diet over the years. Unpublished data from the authors (DG-M, SAC) have shown an increase in feeding raw food in the last five years, which could have affected the cats’ health. Moreover, pet food companies have conducted abundant research in order to improve the quality of their food, which may have been beneficial to the health of pets. For example, it is not uncommon that commercial cat foods are now supplemented with prebiotics and/or probiotics, that can benefit gut microbial populations and reduce pathogens, such as *Escherichia coli*, and so protect from enteric disorders [23,24,25].

About half of the cats (58.2% then 44.3%) were drinking more than previously. This is perhaps not surprising as diseases that result in polydipsia (e.g., chronic kidney disease, hyperthyroidism, and diabetes mellitus) are common in older cats [26,27,28]. Another possibility is that the increased water intake was not genuine but, as with apparently increased food intake, these cats were spending more time indoors, so their drinking was observed more effectively by their owners. Measuring water intake is recommended to establish the significance of any apparent increase. Why the cats in 1995 had a greater increase than in 2010–2015 is unclear. Since the majority of geriatric diseases (including chronic kidney disease and diabetes mellitus) were recognised at a higher prevalence in the 2010–2015 population, the 1995 cats may have been polydipsic as part of an undiagnosed medical condition; alternatively, it may have resulted from more cats in 1995 eating dry cat food and/or more cats in 2010–2015 eating wet food, such that the cats in 1995 needed to drink more water.

Although almost all of the cats had access outside, and most had an indoor litterbox, many of the cats had house-soiled (either urine and/or faeces). When comparing the time points, more cats had access to a litterbox in 2010–2015 (82.9% versus 55.5%), despite that, the prevalence of house-soiling increased (from 29.3% to 55.8%). There are many reasons for house-soiling, including; bladder disease (which in elderly cats most typically results from bacterial urinary tract infections, bladder stones and bladder neoplasia); diseases that result in polyuria and polydipsia (most commonly chronic kidney disease, hyperthyroidism and diabetes mellitus); musculoskeletal diseases (typically arthritis preventing use of cat-flaps and/or high-sided litterboxes); gastrointestinal disease (most commonly chronic enteropathy, alimentary lymphoma or constipation); and behavioural disorders (often due to cognitive dysfunction syndrome (CDS), stress, or organic brain disease—see below) [4,28,29,30,31,32,33,34]. Since there are many treatable or at least manageable conditions that can result in house-soiling, it is important that veterinary surgeons ask about this behaviour, as many owners appear to be too embarrassed to mention it. Since the 2010–2015 questionnaire found more owners supplying an indoor litterbox, it appears that the need for this is now better accepted, although the message always warrants repeating. In addition, just because an owner supplies a litterbox that does not mean it is either accessible or acceptable to the cat; its ease of access, shape, size, contents, and state of cleaning all need to be considered, particularly as in the 2010–2015 questionnaire 35.9% of cats were known to have arthritis. The higher prevalence of house-soiling in 2010–2015 might also be associated with more cats being kept inside, perhaps unwillingly; being housebound makes the observation of this behaviour more apparent, and/or that indoor cats are more stressed. 

Many of the cats had reduced grooming, stopped going outside and now slept most of the time. The reduction in grooming most probably resulted from dental pain, arthritis, CDS and/or hyperthyroidism. The reluctance to go outside may have resulted from difficulties negotiating cat flaps (due to arthritis) or a reduced desire to go outside for other reasons (such as the avoidance of cold weather which will aggravate pre-existing arthritis, or not wanting to encounter other cats as the older cats are no longer able to defend themselves). Given that older cats spend so much time sleeping, it is important that they have easy access to safe, stable, warm, and comfortable beds. In addition, it is important that elderly cats have all key resources easily available, such as food, water, litterbox, resting places (ideally high up but with steps or ramps to facilitate access), hiding places/exit-entry points, etc. [4,35]. The difficulty old cats may have with high-sided litterboxes and/or cat-flaps has already been mentioned.

The cats showed a number of important changes in social behaviour. The most noticeable and consistent of these was the cats becoming more sociable and affectionate towards their owner and more demanding of their owner’s attention. In many cases the cats also became more vocal (63.5%; 58.9%) and cried for attention at night (32%; 43.6%). Other changes included an alteration in their tolerance of other animals they lived with, and generally becoming more tolerant of cats they met outside. These changes are likely a reflection of the cats being aware of their physical limitations as they age, so they know that if they do not tolerate other cats, fighting will likely result in their own injury. Geriatric behavioural changes, including altered interaction with the family and increased vocalisation (particularly at night) are commonly seen in older cats [4,35,36], and can have many different causes, including systemic illness (e.g., hyperthyroidism, hypertension, kidney disease, diabetes mellitus, etc.), organic brain disease, true behavioural disorders, or CDS [33]. A survey of owners of older cats (of 7–11 years of age) revealed that 36% of the cats were showing behavioural problems apparently resulting from CDS, and the frequency of these problems increased with age, such that 88% of cats aged between 16–19 years were affected [37]. A separate study produced similar findings, with 28% of cats aged 11–14 years showing geriatric behavioural changes, increasing to 50% of cats of 15 years and older [38]. The most common behavioural change seen in the 11–14 year old group was alteration in social interactions with people or other pets, while in the 15 years and older group the most common signs were alterations in activity (including aimless walking) and excess vocalisation [38]. A consistent finding from both time points was that over a third of cats were crying at night (32%; 43.6%), having started crying more at a mean of 13 years of age. It is important that veterinary surgeons ask owners about this behaviour as it can be particularly difficult for an owner to live with and may be caused by a number of treatable or at least manageable conditions, including hyperthyroidism, hypertension, and CDS [35,36]. 

The current study appears to show an inverse relationship between the cats’ reduced motivation to go outdoors, hunt or interact with cats outside the house, and an increased dependency on their owners for company, stimulation, and reassurance. It is likely that the lack of desire to go outside results from ill health and/or chronic disability such that the elderly cats find it difficult to get outside and/or have a reduced ability to protect themselves when they are outside. 

Although there were 15 to 20 years between the two surveys, in almost all instances, they had similar findings in relationship to behavioural changes. The findings that differed most between the two time points were the supply of litterboxes and the affection displayed to owners. The proportion of cats with a litterbox increased from 55.5% to 82.9% over time. This appears to reflect that over the 15 to 20−year period more owners appreciated the need for an older cat to have a litterbox (as mentioned above). However, the type of environment these cats lived in was not assessed in either questionnaire, so the increased supply of litterboxes could have resulted from more cats living in apartments in 2010–2015. However, since almost all cats in both time points had outside access, this does not appear to be the case. Another difference was that a higher percentage of owners reported that their cats had become more affectionate in 1995 (51.9%) than in 2010–2015 (35.8%). This was unexpected and is difficult to explain. It could reflect changing owner expectations, with owners in 2010–2015 expecting their cats to be affectionate and so not noting subtle changes in this behaviour, while this was less expected in 1995, so when the cats did show more affection the owners commented on it. Another cause could be over interpretation in 1995, with owners misidentifying behaviours such as increased vocalisation as being a sign of affection, rather than reflective of confusion, attention seeking, deafness or other causes [36]. In comparison, owners in 2010–2015 may have been more aware of these other causes. 

Perhaps not surprisingly, owners reported that many of the cats had health issues. Most of the cats had been taken to see a veterinary surgeon recently, either for routine vaccinations or because of ill health. The high percentage of the cats receiving veterinary care may reflect either their age (older cats have a high risk of developing ill health) [27] and/or the dedication of the owners to the health and welfare of their cats such that they undertook preventative care (e.g., regular examinations and vaccinations). 

Most owners recognised chronic illnesses in their cats. The disabilities reported most frequently in 1995 were arthritis (6.6%), chronic kidney disease (6.1%), and deafness (4.6%); compared to arthritis (35.9%), dental disease (31.3%), and chronic kidney disease (23.1%) in 2010–2015. Interestingly, while owners in 1995 reported that their cats had arthritis and deafness, they only sought veterinary attention for the cats in relation to kidney disease. It is unclear why they did not seek veterinary attention for their cat’s arthritis or deafness. It may have been because the veterinary surgeons did not mention the possibility of these conditions developing during previous consultations, so the owners did not realise that treatment options might be available. The importance of arthritis in older cats should not be overlooked. Radiographic evidence of arthritis is present in 60–90% of cats over 12 years of age, with changes being seen most frequently in elbows, hips, stifles and shoulders [39,40,41,42,43,44]. Unfortunately, while owners appear to be good at recognising arthritis in their cats, and over 90% are willing to have their cat treated for it [45], many veterinary surgeons appear to find arthritis difficult to recognise in cats, so subtleties on historical evaluation, such as toileting habits, become more important. Thankfully, there are now many management options available to reduce pain and improve mobility, so improving the overall quality of life for these cats [39,42,44,46,47].

The apparent prevalence (as determined via owner reporting) of most of the major diseases increased significantly between 1995 and 2010–2015. Notably, these included chronic kidney disease, arthritis, hyperthyroidism, and dental disease. While in most cases this probably resulted from increased clinical awareness, the prevalence of hyperthyroidism may have actually increased, as it did so in this time frame over much of the world [48]. 

There were several associations between behaviours and disease, mainly from the 2010–2015 questionnaire. While most of the behaviours were associated with several different diseases (e.g., kidney disease, blindness, hyperthyroidism, arthritis, and deafness), some of them showed few or even no associations (i.e., heart disease, dental disease, and diabetes mellitus).

Most of the associations were expected, such as increased water intake and weight loss with kidney disease and hyperthyroidism; reduced time spent grooming with dental disease; and less willingness to go outside with arthritis. However, others were not, particularly those associated with deafness and blindness. It is of note that only kidney disease and arthritis were associated with more behavioural changes than these two conditions. This is particularly concerning since owners do not appear to seek veterinary care for arthritis, deafness, or blindness: since these were likely diagnosed by owner assumption rather than veterinary acumen it is possible the owners assumptions, and lack of veterinary investigation, meant that underlying diseases may have been missed, hence the large number of behavioural changes seen with these conditions. While the behavioural changes associated with arthritis were generally expected; many of those for deafness and blindness were not; for example, why would deafness and blindness both be strongly statistically associated (*p* < 0.001) with reduced grooming, increased water consumption, and increased vocalisation (either day or night)? This highlights the importance of recognising normal from abnormal age-related changes. While deafness and blindness may be recognised as signs of normal ageing, they may also be indicative of other underlying conditions or represent more generalised sensory deterioration. This study did not ask owners if they thought their cats were living with dementia (CDS); however, it is of note that mid-life hearing loss appears to be a risk factor of later dementia in humans [49,50]; this is clearly an area that needs more investigation in cats as well as in people.

Previous studies have reported the life expectancy of pet cats is increasing [5,6,7]. However, our findings contradict this, as the median age of the cats was the same in both studies (14–16 years), while the percentage of cats of 20-years old or older was actually higher in 1995 (6.1%) than 2010–2015 (4.3%). This was unexpected. Perhaps the early survey selected for more dedicated owners (see below) who were able to keep their cats alive for longer than the national average, while the second survey was more typical of ‘average’ cat owners. A census-like survey would be needed to investigate further, looking at a very large study population to identify the true percentage of pet cats that live to 20 years or more. 

There are caveats that need to be considered. The owners who completed these surveys may not represent the cat-owning public in general. Given that they were willing to take the time to complete the questionnaires, it is likely that they were more interested in their cat’s health and welfare than all cat owners. With the first survey, the owners had to send away a stamped self-addressed envelope to request a paper copy of the questionnaire, while in the second survey the website is one frequented by particularly feline-focused owners. Because the study populations differed, the validity of their comparison is weaker than would have been the case if the same population could have been sampled on both occasions. In addition, in the second survey the cats’ ages were divided as per the American Association of Feline Practitioners (AAF) and the American Animal Hospital Association (AAHA) [51] so they fitted internationally accepted age groups; however, these differed from those used in the 1995 questionnaire, prohibiting age-group comparison other than for 20 years of age or older. There may also have been recall bias and inherent inaccuracies when asking owners questions about their cat’s health, as few would have been knowledgeable about veterinary medicine. This could have led to miscomprehensions and assumptions, especially with arthritis, deafness, and blindness, which may have been presumed by the owners, rather than diagnosed by their veterinarian. Furthermore, the Vet Professionals website has books and free information on different diseases, such as chronic kidney disease, hyperthyroidism, blindness, and lower urinary tract infections, which might have increased the number of owners reporting these illnesses in the 2010–2015 questionnaire. Owners that had accessed this information would have been in the website’s database, and then invited to complete the survey. However, accepting the limitation that the two time points accessed different owner populations, this paper gives many useful insights into the life of elderly pet cats, their behaviour and health, helping to increase awareness of the needs of this group of cats and to facilitate the provision of more appropriate veterinary care. 

The questionnaire in 1995 shows that owners are very attached to their elderly pet cats. Three quarters of the cats in that study had been owned since kitten-hood, and despite the development of negative behaviours (house-soiling, attention seeking, and excessive vocalisation, especially at night), and the presence of chronic health problems, almost all of the owners (96%) stated that they still gained as much pleasure from having their elderly cat as they had when it was younger. This clearly indicates a strong bond between owners and cats and should provide motivation for owners to modify their homes and lifestyles to benefit their cats, and to invest in veterinary care. Clearly, owners of elderly cats do not want just any cat; they want the cat they already have.

## Figures and Tables

**Table 1:** Increased behavioural changes ^1^. Percentages from both surveys where behavioural changes were reported to be increased by the owners of the elderly cats.

Increased Behaviours	1995	2010–2015
Vocalisation during the day	63.5%	58.9%
Vocalisation at night	32%	43.6%
Sociability with people	51.9%	35.8%
Sociability with animals	25.2%	13%
House-soiling	29.3%	55.8%

^1^ Some of these data have been published previously [11].

**Table 2:** Decreased behavioural changes ^1^. Percentages from both surveys where behavioural changes were reported to be decreased by the owners of the elderly cats.

Decreased Behaviours	1995	2010–2015
Appetite	23.5%	23.5%
Grooming	22.9%	35.6%
Willingness to go outside	52.2%	58.5%

^1^ Some of these data have been published previously [11].

**Table 3:** Prevalence of disease. Percentages from both surveys of the prevalence of different diseases as reported by the owners of elderly cats.

Disease	1995	2010–2015
Arthritis	6.6%	35.9%
Dental disease	0.6%	31.3%
Kidney disease	6.1%	23.1%
Hyperthyroidism	1.2%	13.9%
Deafness	4.6%	12.5%
Blindness	2.5%	10.5%
Lower urinary infections	0.8%	10.4%
Heart disease	0.8%	4.3%

## Supplementary Materials

The following are available online at https://www.mdpi.com/2306-7381/7/3/85/s1, Supplementary Material S1: Questionnaire from 1995, Supplementary Material S2: Questionnaire from 2015, Supplementary Material S3: Table of complete statistical results of logistic regressions.
