# Supplementary material for: Prevalence of Disease and Age-Related Behavioural Changes in Cats: Past and Present
Source: Vet Sci. 2020 Jul 6;7(3):85. doi: 10.3390/vetsci7030085 (PMC7557453; doi:10.3390/vetsci7030085)
Supplement: Supplementary file 1 [file vetsci-07-00085-s001.zip › Supplementary Material 1 S1.pdf]

THE ELDERLY CAT SURVEY

THANK YOU FOR COMPLETING THIS QUESTIONNAIRE. FOR THE PURPOSE OF THIS SURVEY AN ELDERLY CAT IS CONSIDERED TO BE TWELVE YEARS OR OLDER.
(PLEASE TICK THE APPROPRIATE BOX.)

YOUR NAME: ....
ADDRESS: ....
....

POSTCODE: ....
TELEPHONE NUMBER: ....

YOUR CAT'S NAME: .... AGE: YEARS .... MONTHS ......
BREED/TYPE: .... SEX: ☐ MALE ☐ FEMALE
NEUTERED? ☐ YES ☐ NO

HAVE YOU HAD YOUR CAT FROM A KITTEN? ☐ YES ☐ NO
IF NOT, FOR HOW LONG? YEARS .... MONTHS ....

AS YOUR CAT HAS GOT OLDER, HAS HIS/HER APPETITE ...?
☐ INCREASED ☐ DECREASED ☐ STAYED THE SAME

HOW OFTEN DO YOU FEED YOUR CAT?
☐ ONCE A DAY ☐ TWICE A DAY ☐ THREE TIMES A DAY ☐ ON DEMAND

IS YOUR CAT MORE FUSSY ABOUT FOOD THAN WHEN HE/SHE WAS YOUNG?
☐ YES ☐ NO

DOES YOUR CAT DRINK MORE WATER NOW HE/SHE IS OLDER?
☐ YES ☐ NO ☐ DOESN'T DRINK WATER

HOW MUCH DOES YOUR CAT SLEEP IN ONE DAY?
☐ LESS THAN 50% ☐ 50-75% ☐ MORE THAN 75%

DOES YOUR CAT HAVE A FAVOURITE PLACE TO SLEEP? ☐ YES ☐ NO
IF YES, WHERE? ....

DOES YOUR CAT SHARE HIS/HER HOME WITH OTHER ANIMALS? ☐ YES ☐ NO
IF YES, IS YOUR CAT AS SOCIABLE IN HIS/HER OLDER YEARS?
☐ LESS TOLERANT ☐ MORE REMOTE, LIKES TO BE ALONE ☐ MORE SOCIABLE
☐ SAME AS BEFORE

HAS YOUR CAT OUTLIVED A CAT FRIEND IN YOUR HOUSEHOLD? ☐ YES ☐ NO
IF YES, CAN YOU DESCRIBE HIS/HER REACTION TO THE LOSS? ....
....

DOES YOUR CAT GO OUTSIDE?
☐ NO, NEVER HAS ☐ YES, BUT NOT AS MUCH AS WHEN HE/SHE WAS YOUNG
☐ YES, NOW MORE OFTEN NOW HE/SHE IS OLDER ☐ SAME AS BEFORE

WHEN YOUR CAT IS OUTSIDE NOW, DOES HE/SHE STILL HUNT?
☐ HE/SHE HAS NEVER HUNTED ☐ STILL CATCHING PREY ☐ STOPPED COMPLETELY

HOW DOES YOUR CAT VIEW OTHER CATS IN HIS/HER TERRITORY?
☐ AGGRESSIVELY AS ALWAYS ☐ MORE TOLERANT NOW HE/SHE IS OLDER
☐ NEVER FOUGHT WITH OTHER CATS ☐ OTHER ....

HOW HAS YOUR CAT'S ATTITUDE TO YOU CHANGED NOW HE/SHE IS OLDER?

☐ MORE SOCIABLE/AFFECTIONATE ☐ MORE DEMANDING OF ATTENTION

☐ LESS SOCIABLE ☐ OTHER ....

DOES YOUR CAT CALL MORE THAN HE/SHE DID WHEN YOUNGER?

☐ USES MORE SOUNDS TO GET FOOD, ATTENTION ETC.

☐ MIAOWS LESS THAN BEFORE ☐ ABOUT THE SAME

DOES YOUR CAT CALL FOR ATTENTION AT NIGHT? ☐ YES ☐ NO

IF YES, AT WHAT AGE DID THIS BEHAVIOUR START? ....

DO YOU THINK YOUR CAT'S GROOMING HABITS HAVE CHANGED SINCE HE/SHE HAS BEEN OLDER?

☐ STILL GROOMS FREQUENTLY ☐ STILL GROOMS OCCASIONALLY

☐ HAS STOPPED GROOMING

DOES YOUR CAT LIKE TO PLAY WITH TOYS AS MUCH AS WHEN HE/SHE WAS YOUNGER?

☐ STILL PLAYS REGULARLY ☐ DOESN'T PLAY AT ALL

☐ STILL PLAYS OCCASIONALLY ☐ NEVER REALLY PLAYED MUCH

DOES YOUR CAT USE A LITTER TRAY? ☐ YES ☐ NO

HAVE THERE BEEN ANY TOILET ACCIDENTS INDOORS SINCE HE/SHE HAS BEEN OLDER? ☐ YES ☐ NO

HOW MANY TRIPS TO THE VET HAS YOUR CAT HAD SINCE HE/SHE TURNED TWELVE YEARS OF AGE? ☐ NONE ☐ 1-5 ☐ 6-10 ☐ MORE THAN 10

WHAT WERE THE MAIN PROBLEMS ....

....

DOES YOUR CAT SUFFER FROM ANY CHRONIC ILLNESS OR DISABILITY?

☐ YES ☐ NO

IF YES, PLEASE GIVE DETAILS ....

IS YOUR CAT AS MUCH PLEASURE TO YOU NOW HE/SHE IS OLDER?

☐ YES, BECAUSE ....

☐ NO, BECAUSE ....

IF YOU HAVE OBSERVED OTHER BEHAVIOURAL CHANGES IN YOUR CAT NOW HE/SHE IS OLDER OR HAVE A SPECIAL STORY TO RELATE, PLEASE FORWARD THIS ON A SEPARATE SHEET WITH YOUR SURVEY.

☐ PLEASE TICK THIS BOX IF YOU WISH THE CONTENTS OF THIS QUESTIONNAIRE TO REMAIN ANONYMOUS.

PLEASE RETURN TO: VICKY HALLS, CROWS NEST, HENDRAWNA LANE, BOLINGEY, NR PERRANPORTH, CORNWALL TR6 0DG
