# Supplementary material for: Prevalence of Disease and Age-Related Behavioural Changes in Cats: Past and Present
Source: Vet Sci. 2020 Jul 6;7(3):85. doi: 10.3390/vetsci7030085 (PMC7557453; doi:10.3390/vetsci7030085)
Supplement: Supplementary file 1 [file vetsci-07-00085-s001.zip › Supplementary Material 3 S3.docx]

Main statistical results from ordinal and nominal logistic regressions.

| **1995** | **Coefficient** | **SE**  **Coefficient** | **Odds**  **Ratio** | **95% Confidence Interval** | **pvalue** | **Pearson’s chi-square** | **pvalue** |
| --- | --- | --- | --- | --- | --- | --- | --- |
| Time spent sleeping | -0.88 | 0.22 | 0.41 | (0.26, 0.67) | <0.001 | 0.71 | 0.69 |
| Time spent grooming | 0.90 | 0.30 | 2.94 | (1.35, 4.53) | 0.003 | 4.72 | 0.09 |
| Play | 1.36 | 0.22 | 3.91 | (2.50, 6.12) | 0.01 | 0.44 | 0.80 |
| Territorial | 0.19 | 0.13 | 1.21 | (0.94, 1.57) | 0.14 | 0.28 | 0.86 |
| Changes in appetite | 0.39 | 0.09 | 1.48 | (1.24, 1.77) | <0.001 | 13.63 | 0.003 |
| Sociable with other animals | -0.11 | 0.14 | 0.89 | (0.67, 1.17) | 0.398 | 5.74 | 0.12 |
| Willingness to go outside | 0.65 | 0.09 | 1.92 | (1.58, 2.34) | <0.001 | 15.30 | 0.002 |
| Vocalisation during day | -0.50 | 0.10 | 0.60 | (0.49, 0.75) | <0.001 | 8.10 | 0.04 |
| **2010-2015** | **Coefficient** | **SE**  **Coefficient** | **Odds**  **Ratio** | **95% Confidence Interval** | **pvalue** | **Pearson’s chi-square** | **pvalue** |
| Agitation/Irritation | -0.09 | 0.09 | 0.91 | (0.76, 1.09) | 0.32 | 21.65 | 0.02 |
| House-soiling | -0.60 | 0.10 | 0.55 | (0.45, 0.67) | <0.001 | 29.07 | 0.002 |
| Sociable with other animals | 0.21 | 0.09 | 1.24 | (1.03, 1.50) | 0.02 | 8.15 | 0.70 |
| Sociable with people | -0.02 | 0.07 | 0.97 | (0.84, 1.13) | 0.72 | 13.07 | 0.28 |
| Time spent grooming | 0.74 | 0.08 | 2.11 | (1.81, 2.47) | <0.001 | 32.60 | 0.001 |
| Willingness to go outside | 0.73 | 0.08 | 2.08 | (1.76, 2.45) | <0.001 | 46.66 | <0.001 |
| Vocalisation at night | -0.65 | 0.08 | 0.52 | (0.43, 0.62) | <0.001 | 10.58 | 0.47 |
| Vocalisation during day | -0.48 | 0.07 | 0.62 | (0.53, 0.72) | <0.001 | 45.55 | <0.001 |
| Changes in weight | 0.45 | 0.08 | 1.57 | (1.34, 1.84) | <0.001 | 2.66 | 0.75 |
| Time spent sleeping | -0.74 | 0.07 | 0.47 | (0.41, 0.55) | <0.001 | 18.41 | 0.07 |
| Changes in appetite | 0.04 | 0.07 | 1.05 | (0.90, 1.21) | 0.55 | 43.95 | <0.001 |
| Water intake | -0.68 | 0.07 | 0.50 | (0.43, 0.59) | <0.001 | 40.10 | <0.001 |

Main statistical test results from binary logistic regressions.

| **1995** | **Coefficient** | **SE Coefficient** | **Odds**  **Ratio** | **95% Confidence Interval** | **pvalue** | **Hosmer-Lemeshow** | **pvalue** |
| --- | --- | --- | --- | --- | --- | --- | --- |
| Water intake | 0.94 | 0.11 | 2.57 | (2.04, 3.23) | <0.001 | 2.96 | 0.08 |
| Attitude sociable | -0.25 | 0.09 | 0.77 | (0.064, 0.93) | 0.007 | 0.57 | 0.47 |
| Attitude demanding | 0.41 | 0.09 | 1.51 | (1.25, 1.83) | <0.001 | 3.19 | 0.07 |
| Vocalisation at night | 0.34 | 0.09 | 1.40 | (1.15-1.70) | 0.001 | 2.63 | 0.10 |
| House-soiling | 0.82 | 0.10 | 2.29 | (1.87, 2.79) | <0.001 | 0.49 | 0.49 |
| Hunt | -1.12 | 0.13 | 0.32 | (0.24, 0.41) | <0.001 | 0.80 | 0.28 |
| Kidney disease | 0.58 | 0.17 | 1.79 | (1.27, 2.53) | 0.001 | 6.75 | 0.009 |
| Blindness | 0.41 | 0.27 | 1.59 | (0.88, 2.56) | 0.140 | 0.10 | 0.74 |
| Lower urinary tract infection | 0.59 | 0.45 | 1.80 | (0.73, 4.39) | 0.21 | 0.30 | 0.58 |
| Hyperthyroidism | 0.20 | 0.40 | 1.24 | (0.56, 2.74) | 0.59 | 0.12 | 0.73 |
| Diabetes mellitus | -1.54 | 1.02 | 0.21 | (0.02, 1.58) | 0.053 | 3.73 | 0.81 |
| Arthritis | 0.41 | 0.17 | 1.51 | (1.07, 2.12) | 0.02 | 0.11 | 0.74 |
| Heart disease | 0.13 | 0.50 | 1.14 | (0.42, 3.07) | 0.78 | 1.50 | 0.22 |
| Deafness | 1.48 | 0.19 | 4.42 | (3.00, 6.52) | <0.001 | 5.18 | 0.023 |
| **2015** | **Coefficient** | **SE Coefficient** | **Odd**  **Ratio** | **95% Confidence Interval** | **pvalue** | **Hosmer-Lemeshow** | **pvalue** |
| Kidney disease | 0.71 | 0.10 | 2.04 | (1.67, 2.48) | <0.001 | 1.41 | 0.49 |
| Blindness | 0.79 | 0.13 | 2.21 | (1.71, 2.85) | <0.001 | 4.53 | 0.10 |
| Lower urinary tract infection | 0.17 | 0.12 | 1.19 | (0.92, 1.53) | 0.17 | 0.31 | 0.85 |
| Hyperthyroidism | 0.49 | 0.11 | 1.63 | (1.30, 2.05) | <0.001 | 11.28 | 0.004 |
| Diabetes mellitus | 0.41 | 0.25 | 1.51 | (0.91, 2.49) | 0.108 | 0.44 | 0.80 |
| Arthritis | 0.66 | 0.09 | 1.93 | (1.61, 2.32) | <0.001 | 0.41 | 0.81 |
| Heart disease | 0.39 | 0.18 | 1.49 | (1.03, 2.15) | 0.03 | 6.48 | 0.03 |
| Deafness | 1.16 | 0.13 | 3.19 | (2.46, 4.14) | <0.001 | 0.40 | 0.82 |
| Dental disease | 0.22 | 0.08 | 1.25 | (1.05, 1.48) | 0.009 | 3.02 | 0.22 |
